# Therapy and Long-Term Prophylaxis of Vaccinia Virus Respiratory Infections in Mice with an Adenovirus-Vectored Interferon Alpha (mDEF201)

**DOI:** 10.1371/journal.pone.0026330

**Published:** 2011-10-13

**Authors:** Donald F. Smee, Min-Hui Wong, Andrew Russell, Jane Ennis, Jeffrey D. Turner

**Affiliations:** 1 Institute for Antiviral Research, Department of Animal, Dairy and Veterinary Sciences, Utah State University, Logan, Utah, United States of America; 2 Defyrus, Inc., Toronto, Ontario, Canada; French National Centre for Scientific Research, France

## Abstract

An adenovirus 5 vector encoding for mouse interferon alpha, subtype 5 (mDEF201) was evaluated for efficacy against lethal vaccinia virus (WR strain) respiratory infections in mice. mDEF201 was administered as a single intranasal treatment either prophylactically or therapeutically at doses of 10^6^ to 10^8^ plaque forming units/mouse. When the prophylactic treatment was given at 56 days prior to infection, it protected 90% of animals from death (100% protection for treatments given between 1–49 days pre-infection), with minimal weight loss occurring during infection. Surviving animals re-challenged with virus 22 days after the primary infection were protected from death, indicating that mDEF201 did not compromise the immune response against the initial infection. Post-exposure therapy was given between 6–24 h after vaccinia virus exposure and protection was afforded by a 10^8^ dose of mDEF201 given at 24 h, whereas a 10^7^ dose was effective up to 12 h. Comparisons were made of the ability of mDEF201, given either 28 or 1 day prior to infection, to inhibit tissue virus titers and lung infection parameters. Lung, liver, and spleen virus titers were inhibited to nearly the same extent by either treatment, as were lung weights and lung hemorrhage scores (indicators of pneumonitis). Lung virus titers were significantly (>100-fold) lower than in the placebo group, and the other infection parameters in mDEF201 treated mice were nearly at baseline. In contrast, viral titers and lung infection parameters were high in the placebo group on day 5 of the infection. These results demonstrate the long-acting prophylactic and treatment capacity of mDEF201 to combat vaccinia virus infections.

## Introduction

The threat of using orthopoxviruses, variola and monkeypox, as bioterror weapons has led to increases in vaccination particularly in military personnel as well as the investigation of countermeasures (antiviral treatments) for such infections [1]. A number of compounds have been identified that exhibit direct antiviral activity against these and related poxviruses in animal models [2], [3]. From these investigations, three compounds stand out as being clinical candidates, cidofovir [4], CMX001 (an orally active prodrug of cidofovir) [5], and ST-246 [6]. Cidofovir, CMX-001, and ST-246 have all been used in emergency settings to treat vaccination complications [7], [8].

Immune system stimulation via exogenous recombinant interferon (rIFN) is effective for treating vaccinia virus respiratory infections in mice [9]. Single daily doses of rIFNα or rIFNγ rescued mice from lethality when administered 1–2 days after virus challenge and reduced lung virus titers. Both vaccinia keratitis and vaccinia-induced skin lesions were treated with rIFN (topically and injected respectively) in monkey models with success [10], [11]. Vaccinia and other poxviruses have evolved comprehensive mechanisms to antagonize the interferon system [12], that include blocking IFN gene induction, disrupting extracellular and intracellular signaling and slowing IFN induced gene (PKR and 2′5′ OAS) activation. Treatment with exogenous rIFN only compensates for host IFN gene suppression and swamping the IFN binding protein so it must begin early after infection before the virus suppresses activation of the antiviral state. Interferon has a short half-life of three hours [13], which requires frequent treatment with large bolus doses resulting in commensurate adverse effects often leading to patient-initiated cessation of treatment [14], [15]. Pegylated rIFN has improved the in vivo half-life to allow for weekly injections, but results in a reduced activity of the protein and an increased cost.

A needle-free, single dose drug capable of achieving steady-state method of delivering interferon would maximize the therapeutic benefits of IFN, while minimizing the bolus-induced toxicity. To this end, a replication-deficient adenovirus 5 (Ad5) vector containing the human consensus interferon alpha gene, referred to as DEF201, has been developed. Intranasal administration of DEF201 allows for the Ad5 vector to infect respiratory cells and drives constitutive expression of the IFN transgene and secretion of fully glycosylated IFN. Ad5-vectored mouse interferon (mDEF201) resulted in sustained IFN levels [16], that completely protected mice from a lethal Western equine encephalitis virus infection when given intramuscularly at 10^7^ plaque forming units (PFU)/mouse up to 7 days prior to virus challenge [16]. Against Venezuelan equine encephalitis virus infection, mDEF201 prevented death when administered intramuscularly at 10^7^ PFU/mouse 24 h prior to infection but not when given 6 h after infection [17]. Intranasally-administered mDEF201 was also used to treat mice infected intranasally with SARS virus (resulting in a lethal respiratory infection) [18]. It was 100% protective when administered prophylactically at 10^6^ PFU/mouse up to 14 days pre-virus exposure, with similar protection afforded by a 10^8^ dose administered therapeutically at 12 h after infection [18].

Adenoviral vectored human consensus IFN (DEF201), was recently used intranasally to treat hamsters infected intraperitoneally with yellow fever virus (resulting in a lethal hepatic infection) [19]. Protection from death was observed for single administrations of DEF201 at 10^7.5^ PFU/animal given 7 days before to 2 days after virus challenge, which demonstrated both prophylaxis and therapy in this model. DEF201 treatment reduced yellow fever virus titers in liver and spleen, and resulted in decreases in serum alanine transaminase levels.

Herein, mDEF201 was evaluated for prophylaxis and therapy of vaccinia virus (WR strain) respiratory infections in mice. In this model the virus infection spreads systemically to include other tissues besides the lungs, particularly the liver and spleen [20]. Increases in lung weight and lung hemorrhage (indicative of pneumonitis) are hallmarks of vaccinia infection, as is severe body weight loss. The results show that a single intranasal dose of mDEF201 produced a remarkably prolonged 8-week prophylactic effect, and was also active therapeutically.

## Materials and Methods

### Ethics statement

The experiments were conducted in accordance with Protocol 552 approved by the Institutional Animal Care and Use Committee of Utah State University. The work was done in the AAALAC-accredited Laboratory Animal Research Center of Utah State University in accordance with the National Institutes of Health Guide for the Care and Use of Laboratory Animals (Revision; 2010).

### Animals

Female BALB/c mice (Charles River Labs, Wilmington, MA) weighing approximately 13–15 g at the time of first treatment were used. After a 48-hour quarantine period the animals were randomly assigned to cages. All work with these animals was performed in the Biosafety Level 2 area of the AAALAC-accredited Laboratory Animal Research Center at Utah State University.

### Test materials

The adenovirus vectored mouse interferon alpha, subtype 5 construct mDEF201 was prepared at the Robert Fitzhenry Vector Laboratory (McMaster University, Hamilton, Canada). The methods of preparation have been described previously [16]. Briefly, the mouse interferon alpha gene (subtype 5) was cloned into a replication deficient Ad-5 vector (deletions of E1 and E3 gene deletions), amplified in 293 cells and purified by cesium chloride gradient centrifugation. Stock solutions of mDEF201 were stored at −80°C and were thawed on ice and diluted in saline to the appropriate dose immediately prior to a single administration. Gilead Sciences (Foster City, CA) provided the positive control compound cidofovir.

### Virus

Vaccinia virus (WR strain) was purchased from the American Type Culture Collection (ATCC, Manassas, VA). The virus was propagated in African green monkey kidney (MA-104) cells (from MA Bioproducts, Walkersville, MD) for use in these studies.

### Experimental design for animal studies

Mice were anesthetized with ketamine/xylazine (50/5 mg/kg) by intraperitoneal (i.p.) injection for intranasal treatments and intranasal infection. The animals were initially infected intranasally with approximately 1–2×10^5^ PFU of vaccinia virus in a 50-µl volume. mDEF201 was administered intranasally (50-µl) either prior to or after vaccinia virus exposure, according to the experimental schedule. Placebo-treated mice were given saline. In order to maintain consistency since intranasal treatment alters the environment for infection, all mice were given intranasal saline when the mDEF201 treatments were given 1 day before or after virus infection, or else additional placebo groups were used to accommodate delivery of intranasal liquid. All animals were weighed every other day. In some instances a normal control group (untreated, uninfected) of 10 mice was maintained throughout the study.

For certain studies, the normal control group was infected with virus at the end of the initial 21-day antiviral study to serve as naïve, infected controls for the re-infection. Treated mice surviving the primary infection were re-infected on day 22 with approximately 5×10^5^ PFU units of vaccinia virus in a 50-µl volume. This dose was larger than for the primary infection to account for the increased age of the mice, and older mice are less susceptible to vaccinia infection. The age-matched normal control (naïve) animals were infected for the first time with the same virus challenge. Survival and body weights in these groups were determined over a 21-day period.

Separate animals were maintained for determination of tissue virus titers, lung hemorrhage scores, and lung weights. Groups of 5 infected mice per day were sacrificed for removal of tissues. Lungs were given a hemorrhage score (color change from pink to plum which occurs regionally in the lung rather than gradually over the entire lung) ranging from 0 to 4 (entire lung affected). Lungs, spleens, livers, and snouts were weighed prior to homogenization which releases infectious virus for titration. Homogenization of soft tissues was done in 1 ml of cell culture medium using a stomacher. Snouts were ground in 1 ml of medium using sterilized mortars and pestles. Samples were serially diluted in 10-fold increments and plaque titrated in 12-well microplates of Vero cells. Plaques were stained at three days with 0.2% crystal violet in 10% buffered formalin, then the plaques were counted with the aid of a light box. Plaque numbers were converted to PFU per gram of infected tissue.

### Statistical analysis

Initially, survivor numbers were compared by multiple group chi square analysis. When statistical significance was found, survivor numbers were evaluated using the two-tailed Fisher exact test. Differences in the mean day of death, tissue virus titers, lung weights, and lung hemorrhage scores were statistically analyzed using the two-tailed Mann-Whitney U-test. Analyses were performed using the InStat® computer software program (GraphPad Software, San Diego, CA), comparing treated and placebo groups.

## Results

### Short-term mDEF201 prophylaxis is completely protective against lethal challenge

The short-term prophylactic activity of mDEF201 administered at various intranasal doses one day prior to virus challenge is presented in Table 1. mDEF201 was uniformly, 90–100% protective from a lethal vaccinia challenge at doses of 10^5^ to 10^7^ PFU/animal. This protective effect can be attributed to the mIFN transgene because the empty adenovirus vector was not protective and produced similar complete mortality as the placebo control. Cidofovir (positive control compound) was also 100% protective at 100 mg/kg/day when administered by intraperitoneal route starting 1 day post-infection. Table 1 also shows the effects of treatment with mDEF201 and cidofovir on lung infection parameters. A dose-responsive effect on lung virus titer was seen with mDEF201 treatments, with the highest dose causing nearly an 800-fold reduction in lung virus titer. mDEF201 doses of 10^5^ and 10^6^ PFU/mouse reduced virus titers 8- and 125-fold, respectively. All three mDEF201 treatments significantly reduced lung weights and lung hemorrhage scores. In contrast, cidofovir treatment caused a only a 40-fold reduction in virus titer, and protected lungs from virus-induced pathology. Lung virus titers and lung weights were also intermediate between the 10^5^ and 10^6^ mDEF201 dosage groups. Neither the control, nor adenovirus empty vector inhibited lung infection parameters.

**Table 1:** Effects of treatment with mDEF201 and cidofovir on survival of mice from a vaccinia virus (WR strain) respiratory infection.

				Lung Infection Parameters (Day 5)		
Compound (dose)^a^	Treatment StartTIme^b^	Survivors/Total	MDD^c^ ± SD	HemorrhageScore	Weight (mg)	Virus Titer (Log_10_ PFU/g)
mDEF201 (10^7^)	−24 h	10/10***	-	0.0±0.0**	128±13**	5.8±0.6**
mDEF201 (10^6^)	−24 h	10/10***	-	0.0±0.0**	130±12**	6.6±1.0**
mDEF201 (10^5^)	−24 h	9/10***	13.0	0.1±0.2**	188±19*	7.8±0.1**
Cidofovir (100)	+24 h	10/10***	-	0.0±0.0**	146±11**	7.1±0.2**
Ad-5 empty vector (10^7^)	−24 h	0/10	6.8±0.6	1.0±0.5	260±58	8.7±0.1
Placebo	−24 h	0/20	7.1±1.0	1.8±0.3	310±66	8.7±0.1

a mDEF201 and empty vector are PFU/mouse doses, whereas the dose of cidofovir is mg/kg/day. mDEF201, the Ad-5 empty vector, and the placebo groups were given single intranasal treatments. Cidofovir was administered by intraperitoneal route once a day for two days. For consistency with the other groups, the cidofovir group also received saline intranasally at −24 h.

b Relative to intranasal virus exposure (1×10^5^ PFU).

c Mean day of death of animals that died on or before day 21 of the infection.

*P<0.05.

**P<0.01.

***P<0.001, compared to placebo.

### Extended prophylaxis of vaccinia infection with a single mDEF201 administration

**Figure 1:**
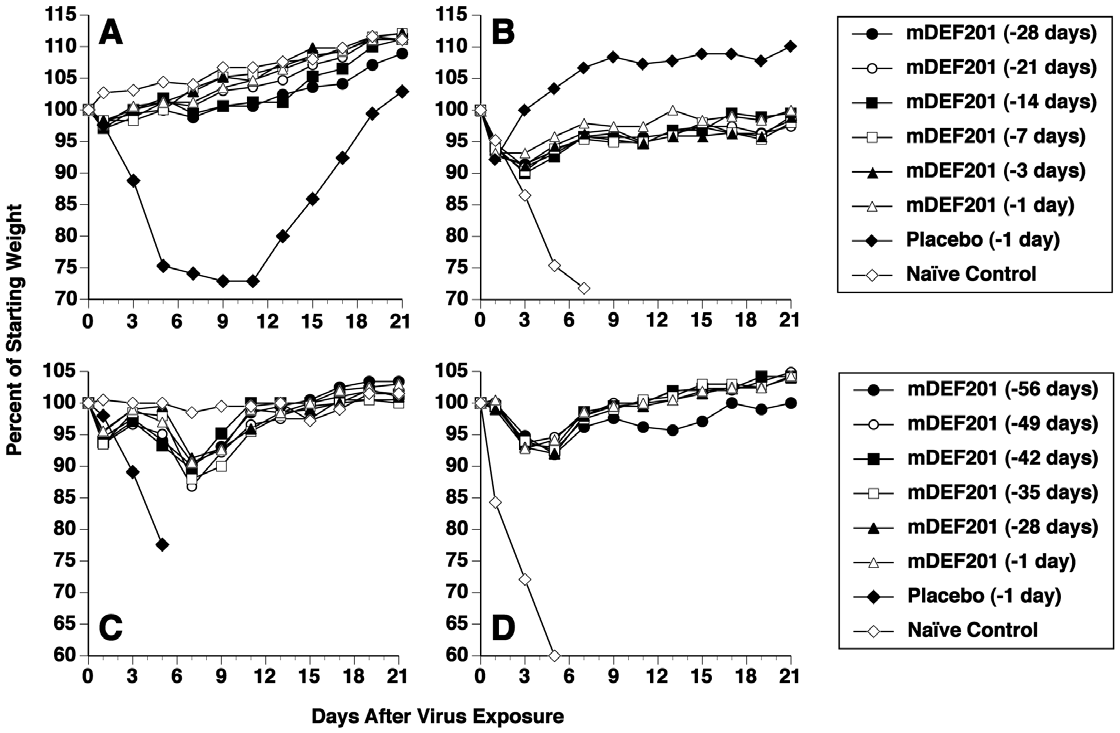
Effects of prophylaxis with mDEF201 on percent body weights of mice during vaccinia virus (WR strain) respiratory infections. (A) Experiment 1, primary infection, (B) Experiment 1, reinfection, (C) Experiment 2, primary infection, (D) Experiment 2, reinfection. Intranasal treatments with mDEF201 (10^7^ PFU/mouse) were given one time only on the indicated day prior to virus exposure. In Experiment 1 the primary intranasal challenge was 1×10^5^ PFU of virus. Since this challenge dose failed to cause 100% mortality (as mice age their susceptibility to infection wanes), the primary virus challenge dose was increased to 2.5×10^5^ PFU of virus for Experiment 2. Reinfection of both sets of mice was performed intranasally with 5×10^5^ PFU of virus. The data accompany those of Table 2. Thus, the initial number of mice per group for each figure corresponds to the total number per group in the table.

To measure the window of prophylactic activity, 10^7^ PFU/mouse of mDEF201 was administered at various times prior to virus infection (Table 2). In the first experiment a single dose of mDEF201 was 100% protective when administered between 28 days and 1 day pre-virus exposure. Mean body weights during the course of the primary infection are presented in Figure 1A. All of the treatments with mDEF201 largely protected the animals against weight loss. A slight drop in weight occurred on day 1 following intranasal exposure to virus, but the mice were back to the initial starting weight by day 3. Seven placebo-treated animals survived the infection, differing from that of Table 1 where no animals survived. This likely occurred because the mice at the time of infection were 28 days older, and susceptibility of the animals to infection diminishes with age. Severe weight loss in the placebo group persisted until day 11 of the infection. After that time, the seven animals that survived the infection began to recover body weight.

**Table 2:** Effects of extended prophylaxis with mDEF201 on survival of mice from primary respiratory infection and reinfection with vaccinia virus (WR strain).

Compound^a^	TreatmentStart Time^b^	Primary InfectionSurvivors/Total	ReinfectionSurvivors/Total
Experiment 1:			
mDEF201	−28 days	10/10**	10/10***
mDEF201	−21 days	10/10**	10/10***
mDEF201	−14 days	10/10**	10/10***
mDEF201	−7 days	10/10**	10/10***
mDEF201	−3 days	10/10**	10/10***
mDEF201	−1 day	10/10**	10/10***
Placebo	−1 day	7/20	7/7***
Naïve Control^c^	-	10/10	0/10
Experiment 2:			
mDEF201	−56 days	9/10***	9/9***
mDEF201	−49 days	10/10***	10/10***
mDEF201	−42 days	10/10***	10/10***
mDEF201	−35 days	10/10***	10/10***
mDEF201	−28 days	10/10***	10/10***
mDEF201	−1 day	10/10***	10/10***
Placebo	−1 day	0/20	-
Naïve Control^c^	-	10/10	0/10

a The mDEF201 intranasal dose of 10^7^ PFU/mouse was given only once. All animals not receiving mDEF201 on day -1 were treated intranasally with saline on that day.

b Relative to the primary infection. In Experiment 1 the primary intranasal challenge was 1×10^5^ PFU of virus. Since this challenge dose failed to cause 100% mortality (as mice age their susceptibility to infection wanes), the primary virus challenge dose for Experiment 2 was increased to 2.5×10^5^ PFU. Reinfection of both sets of mice was performed intranasally with 5×10^5^ PFU of virus.

c Uninfected, untreated for the primary infection, but infected at 22 days.

**P<0.01.

***P<0.001, compared to placebo (primary infection) or naïve control (reinfection).

The surviving mice from experiment 1 were re-infected with a 5-fold higher challenge of vaccinia virus than was originally given, and all of these animals survived the re-challenge (Table 2). Infection of age-matched naïve mice resulted in expected 100% mortality. Weight loss occurred in all mDEF201 treated groups, but was much less severe than in naïve, infected mice (Figure 1B). Rebound from weight loss during the re-infection was fastest in the placebo group. This group contained the most seriously afflicted animals during the primary infection, thus would be expected to have a more robust immune response than treated animals that did not get as sick.

Based upon the successful extended prophylaxis up to 28 days pre-infection, a second experiment (Table 2) was initiated using starting times as early as 56 days before virus challenge. mDEF201 was 90% protective when administered at -56 days, and 100% protective when given at all shorter time points (<49 days). Because the virus challenge dose was higher than that given in Experiment 1 (see footnote “b” in Table 2), the result was that all placebo-treated animals in Experiment 2 died from the primary infection. Minimal weight loss occurred in groups treated prophylactically with mDEF201 (Figure 1C). Decreases in body weight in mDEF201 groups were primarily seen on days 5–9 of the infection, with the nadir being day 7.

The surviving mice that were treated with mDEF201 in the second experiment (Table 2) were re-infected with a 2-fold higher challenge of vaccinia virus, and all of these animals survived. Infection of age-matched naïve mice resulted in 100% mortality. Weight loss occurred in all mDEF201 treated groups, but was minimal and occurred on days 3–5 (Figure 1D). This is in contrast to the rapid weight decline in naïve, infected mice. Body weights during the re-infection were similar for most mDEF201 groups. Slightly less rapid recovery in body weight over 21 days was seen in the mDEF201 group treated prophylactically at -56 days compared to the other treated groups.

**Figure 2:**
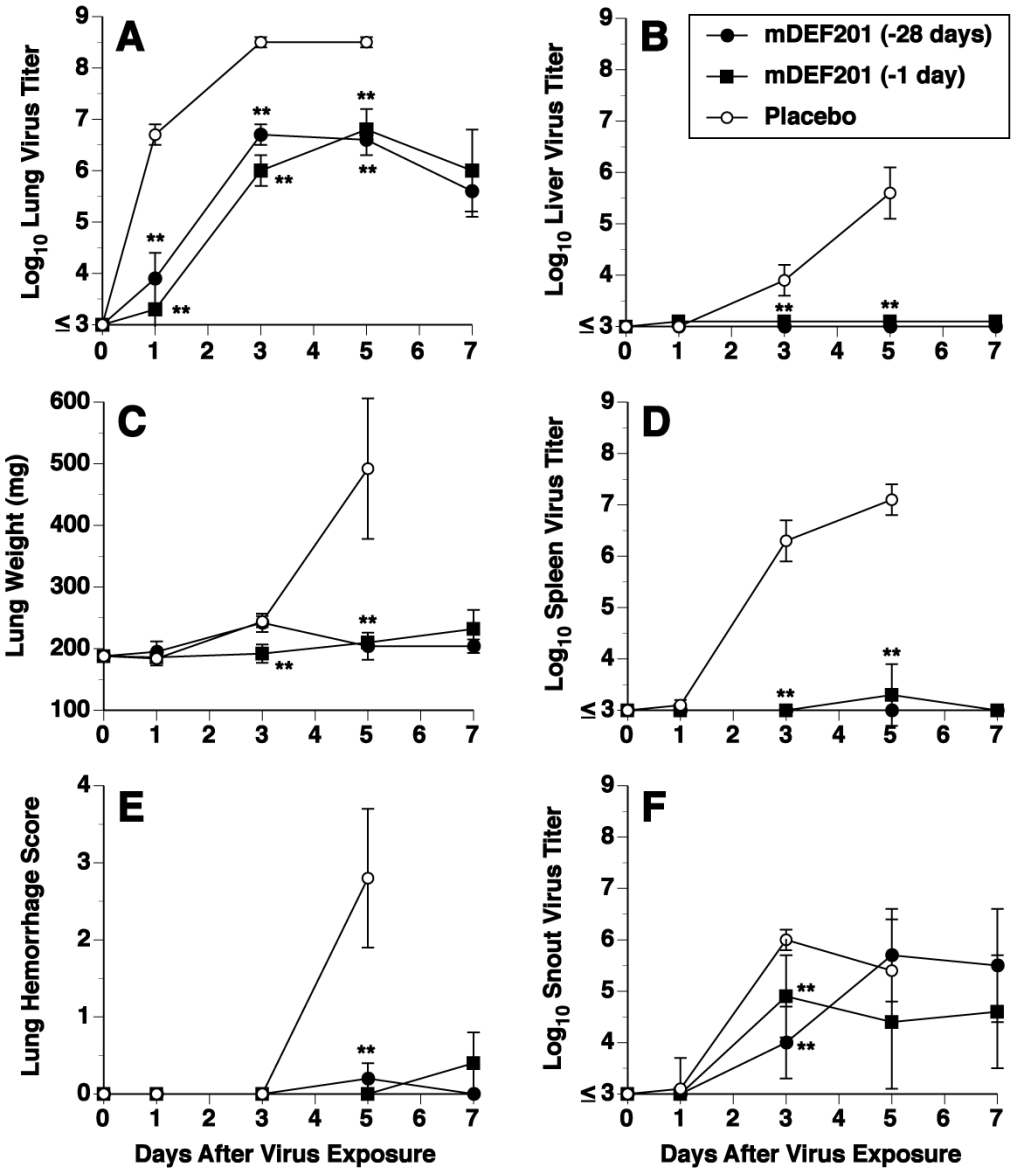
Effects of prophylaxis with mDEF201 given on day -28 or day -1 on infection parameters in mice infected intranasally with vaccinia virus (WR strain). (A) Lung virus titer, (B) liver virus titer, (C) lung weight, (D) spleen virus titer, (E) lung hemorrhage score, (F) snout virus titer. Intranasal treatments with mDEF201 (10^7^ PFU/mouse) were given one time only on the indicated day prior to intranasal virus exposure (2.5×10^5^ PFU). There were no survivors in the placebo group on day 7 for data determinations. This experiment was conducted concurrently with the second experiment in Table 2. Each data point represents the mean for five animals per group.

Viral titers in various tissues were determined from mice treated with mDEF201 either at 28 days or 1 day prior to infection. Lung virus titers in mDEF201 treated groups were at least 100-fold less than in placebo treated mice (Figure 2A). Liver and spleen virus titers were below or near the level of detection in animals treated with mDEF201, but increased over time in placebos (Figures 2B and 2D). Snout virus titers in mDEF201 treated groups were significantly less than placebos on day 3 but not on day 5 (Figure 2F). In comparing virus titers in the -28 day mDEF201 group to that of the -1 day mDEF201 group, the amounts of detectable virus were nearly equivalent in lung, liver and spleen. Differences were observed in snout virus titers between these two groups, but due to variability the differences were not statistically significant.

Lung weights and lung hemorrhage scores were determined in infected mice in parallel with the determination of viral titers. Lung weights in mDEF201 treated animals were near normal, whereas on day 5 the placebos exhibited a large increase in lung weight (Figure 2C). Similarly, lung scores were near normal in the two groups treated with mDEF201, but were severe on day 5 in the placebo group (Figure 2E). The extent of inhibition of lung disease in the -28 days mDEF201 group was comparable to that seen in the -1 day mDEF201 group.

The results from these experiments indicate that mDEF201 has an extremely long-acting prophylactic activity against vaccinia virus infections in mice. Only in animals treated 56 days prior to infection was there a hint of waning activity, since one animal died from the infection in that group.

### Therapy of infection with mDEF201

mDEF201 at two different doses was administered after infection to combat a vaccinia virus infection. Preliminary studies with low dose mDEF201 (10^5^ or 10^6^ PFU/mouse), given at 6, 12, or 24 h after infection provided no protection from the lethal infection (data not shown). However, higher doses of mDEF201 (10^8^ PFU/mouse) were 100% protective when administered either at 6, 12, or 24 h after infection (Table 3). A 10^7^ PFU/mouse dose was 80–90% protective when given at 6 and 12 hours, but only 30% protective when administered at 24 hours. Survival time in the lower dosage group receiving treatment at 24 hours was significantly increased relative to the placebo control. Cidofovir was 100% protective when administered at 100 mg/kg/day for two days starting at 24 h.

**Table 3:** Effects of therapy with mDEF201 and cidofovir on survival of mice from a vaccinia virus (WR strain) respiratory infection.

Compound(Dose)^a^	TreatmentStart Time	Survivors/Total	MDD^b^ ± SD
mDEF201 (10^8^)	+6 h	10/10***	-
mDEF201 (10^7^)	+6 h	9/10***	8.0
Placebo	+6 h	0/10	6.8±0.6
mDEF201 (10^8^)	+12 h	10/10***	-
mDEF201 (10^7^)	+12 h	8/10***	9.0±2.8
Placebo	+12 h	0/10	7.1±0.3
mDEF201 (10^8^)	+24 h	9/9***	-
mDEF201 (10^7^)	+24 h	3/10	9.6±2.8**
Cidofovir (100)	+24 h	10/10***	-
Placebo	+24 h	0/10	7.1±0.3

a The doses of mDEF201 are in PFU/mouse units, whereas the dose of cidofovir is in mg/kg/day units. mDEF201 was given as a single intranasal treatment, as was the placebo Cidofovir was administered by intraperitoneal route once a day for two days. For consistency with the other groups, the cidofovir group also received intranasal saline at 24 h.

b Mean day of death for mice that died on or before day 21 of the infection. The mice were inoculated intranasally with 1×10^5^ PFU of virus to initiate the infection.

**P<0.01.

***P<0.001, compared to placebo.

**Figure 3:**
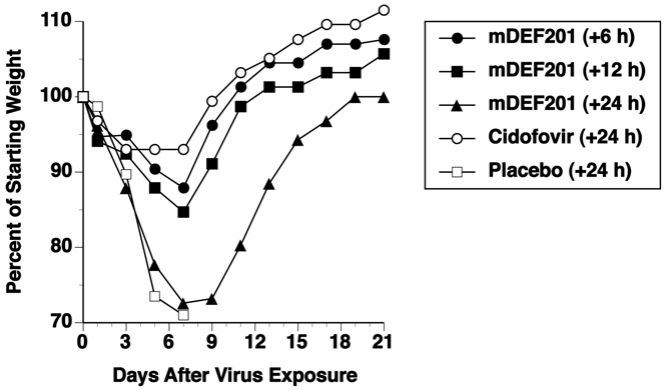
Effects of therapy with mDEF201 and cidofovir on percent body weights of mice during a vaccinia virus (WR strain) respiratory infection. Intranasal treatments with mDEF201 (10^8^ PFU/mouse) were given one time. Cidofovir (100 mg/kg/day) was administered by intraperitoneal route once a day for two days. Test materials were given at the time indicated after intranasal virus exposure (1×10^5^ PFU). The data accompany those of Table 3 (body weight results of treatment with mDEF208 at 10^7^ PFU/mouse are not shown). Thus, the initial number of mice per group for each figure corresponds to the total number per group in the table.

Body weight changes for treatments with 100% survival during the course of the infection are shown in Figure 3 (10^8^ mDEF201 and cidofovir). Weight loss in mDEF201 treated mice increased with longer delay to commencement of treatment, with all animals regaining their pre-challenge weight by day 18 of the experiment. Given the weight loss in the +24h group, further delays in treatment would likely result in some mortality. Mice treated daily with cidofovir starting 24h post-vaccinia challenge lost less weight.

## Discussion

In these studies, a single intranasal dose of mDEF201 was found to exhibit a potent prophylactic activity that endured for at least 56 days. An endpoint in the duration of the protection could not be projected because treatment at -56 days resulted in only one death and caused minimal weight loss during the infection. Indeed, weight loss during infection for the -56 day treatment group was equivalent to the -1 day treatment group. Investigation of other parameters of infection (tissue virus titers, lung weights, and lung hemorrhage scores) revealed that the inhibition of these parameters with a -28 day mDEF201 treatment was quite comparable to that of a -1 day treatment. Prophylaxis starting a day before infection could effectively be achieved with doses of 10^5^ through 10^7^ PFU/mouse. Extended prophylaxis up to 56 days pre-infection was only investigated at the 10^7^ dose.

Other investigators have shown that DEF201 in both mouse and human constructs are active prophylactically [16], [17], [18], [19]. Kumaki et al. showed 100% protection against SARS infection in mice by a 10^6^ dose given only at -14 days (other time points were not assessed) [17]. Thus, it is possible that prophylaxis earlier than 14 days would be protective against SARS infection. Against yellow fever virus infections in hamsters, DEF201 was effective when given 7 days prior to infection but not at -21 days [19]. This breadth of viral efficacy indicates the potential of DEF201 to function as a truly broad-spectrum antiviral.

Remarkably, mDEF201 was effective in mice against vaccinia virus infection when given nearly two months prior to virus exposure. This indicates that Ad5-vectored IFN induced a long-term antiviral state that was completely protective with a single dose. This extended prophylaxis appears to exist in the absence of measurable serum IFN levels, since Wu et al. [15] were only able to detect interferon alpha protein in mice treated intramuscularly with mDEF201 at 24 and 48 h, but not at 7 days (times in between were not investigated).

This study raises a number of important questions relative to the long acting protective effect of mDEF201 against vaccinia virus infection in mice. Questions such as how much interferon is produced, how long is it produced, and what cells are responsible for producing it are being asked and are currently under investigation. It has already been shown that interferon is successfully produced by the vector when administered intramuscularly to mice and the protein is detectable in the serum [16]. From that work we also know that the levels of interferon rise very quickly (within 3-5 h), are transiently high, and come down within several days. We can infer that the interferon protein will be delivered successfully to the lung and nostrils (and have since confirmed this in a separate study, unpublished). Given that the protection lasts longer than the previously measured levels of interferon protein persist, we assume that the interferon is activating an immunological cascade that then protects the animal from infection by inducing an antiviral state. Thorough investigation of induced genes, length of induction, and localization of induction are planned, but are beyond the scope of this study.

The choice of intranasal route of administration is important, as this route has been demonstrated to bypass pre-existing immunity to the adenovirus vector [21]. Intranasal delivery is also less invasive and easier to administer to large numbers of patients in the event of a mass infection due to a natural outbreak or intentional release. Adenovirus was selected to be the vector for interferon due to its rapid infectivity, breadth of human safety data, and facility of intranasal administration. mDEF201, administered by intranasal route, has already been shown to be effective in the treatment of SARS coronavirus respiratory infections in mice [18]. This is the first description of adenoviral vectored interferon alpha being used, by any route, to prevent or treat vaccinia virus infections.

A limited therapeutic window of time exists for treating vaccinia virus infections with interferon-based drugs like mDEF201, and to achieve treatment higher doses of mDEF201 are needed than for prophylaxis. Indeed, low dose mDEF201 (10^5^–10^6^ PFU/mouse) that were effective prophylactically were not effective when administered even 6 h after infection. Higher doses (10^8^ PFU/mouse) were required for full protection from lethality when given at +24 h, however considerable body weight loss resulted due to infection (Figure 3). The single 10^8^ dose administered at 24 h was not as effective as daily doses of 100 mg/kg of cidofovir that acts directly on vaccinia to inhibit replication. This may be due in part to the lag time between administration of mDEF201 and the production of therapeutic levels of IFN within 6 h [15]. Secondly, in situ produced IFN must overcome a high level of IFN system down regulation caused by the established vaccinia infection [12]. It has been demonstrated by other investigators that both mDEF201 and DEF201 are effective as a treatment only when given within 1–2 days after virus exposure [16], [17], [18], [19]. How well Ad-vectored IFN protects infected animals will depend upon each virus' virulence including the number and expression of different IFN system antagonists. However, the treatment efficacy of DEF201 in these unrelated viruses indicates real potential as a broad spectrum antiviral, and the extrapolation of these treatment windows into the clinical scenario may be significant.

In these studies antiviral activity depended on the amount of mDEF201 administered, which was the case in other published studies with unrelated viruses [16], [17], [18], [19]. The production of a steady-state level of interferon may bypass the need for bolus dosing associated with traditional interferon treatment, and thus reduce toxic side effects. Nevertheless, safety of DEF201 is a consideration for eventual human use, and safety and toxicology studies are ongoing. It will need to be determined how long the IFN-induced antiviral state persists and its effects on treated individuals.

This is the first study demonstrating that surviving mice treated with mDEF201 are protected against re-infection with the same virus. This is not surprising, as mDEF201 treatment did not completely prevent vaccinia virus production in the lungs and snouts of the animals (Figures 2A and 2F), thus driving an acquired immune response. The veracity of that immune response in mDEF201 treated animals most likely was weaker than it would be in more severely infected mice, based upon weight comparisons to surviving placebos (Figure 1B).

Assuming an adequate safety profile, one can envision the use of DEF201 as a means of quickly providing significant protection to first responders and medical chain personnel confronted with a deliberate release of variola or monkeypox virus into the environment. Even if infected, DEF201 treated individuals could still acquire an immunity to the virus, as demonstrated by the present studies, thus rendering them ‘vaccinated’ against future exposure. The intranasal method of delivery of DEF201 facilitates rapid prophylaxis in people entering a suspected poxvirus environment. Moreover, DEF201 has the potential to treat a large number of unrelated viruses simultaneously, for which there are no current treatments.
